# Sertoli - Leydig cell tumor with retiform areas and overgrowth of rhabdomyosarcomatous elements: case report and literature review

**DOI:** 10.1186/s13048-016-0257-4

**Published:** 2016-07-30

**Authors:** T. Burnik Papler, S. Frković Grazio, B. Kobal

**Affiliations:** 1 Division of Obstetrics and Gynecology, University Medical Centre Ljubljana, Slajmerjeva 3, 1000 Ljubljana, Slovenia; 2 Department of Gynecological Pathology, Division of Obstetrics and Gynecology, University Medical Center Ljubljana, Ljubljana, Slovenia; 3 Department of Obstetrics and Gynecology, University Medical Centre Ljubljana, Slajmerjeva 3, 1000 Ljubljana, Slovenia; 4 Department of Gynecological Pathology, University Medical Centre Ljubljana, Slajmerjeva 2, 1000 Ljubljana, Slovenia

**Keywords:** Sertoli - Leydig cell tumor, Retiform, Rhabdomyosarcoma, Postmenopausal woman

## Abstract

**Background:**

Sertoli - Leydig cell tumors (SLCTs) are sex-cord stromal tumors that account less than 0.5 % of primary ovarian neoplasms. They are mostly benign and occur in reproductive age women. Variants with heterologous mesenchymal elements are exceptionaly rare. The usual presentation of SLCTs is with signs of androgen excess as majority of them produce androgens.

**Case presentation:**

We present a case of a SLCT occurring in a 70 year old woman. Her presenting complaint was abdominal distension and pain. She had no signs of androgen or estrogen excess. Transvaginal ultrasound (TVUS) and CT scan showed a multilocular adnexal tumor and level of CA 125 was raised. A complete cytoreduction was achieved with surgical procedure. Histopathological examination revealed moderately differentiated SLCT with retiform areas and owergrowth of heterologous component in form of embrional rhabdomyosarcoma (RMS). She returned 7 months after the surgery with a large abdominal mass, ascites, right- sided hydronephrosis and massive pulmonary embolism. Due to the widespread disease and her poor general condition, she received only palliative care. She died 15 days after the admission. No autopsy was performed.

**Conclusions:**

Due to the rarity of SLCTs, especially those with retiform areas and heterologous elements, their management remains challenging. There is no firm evidence that adjuvant chemotherapy is effective in improving survival in SLCTs with malignant heterologous elements. Further studies with a higher number of cases and a longer follow-up are needed to better predicting the prognosis and determine the role of chemotherapy in such cases.

## Background

Sertoli – Leydig cell tumors (SLCT) are sex cord-stromal tumors that account for less than 0.5 % of primary ovarian neoplasms [1]. Because of their scarcity SLCT represent a challenge in diagnosis and management.

The vast majority of SLCT (90 %) occur in the reproductive age and the rest before menarche or after menopause [2]. They are usually unilateral and confined to the ovary at the time of diagnosis. Approximately 80 % of SLCT are hormonally active with elevated serum testosterone and androstenedione levels and one third of these tumors is discovered due to the clinical signs and symptoms of excess androgen production (virilization, voice deepening, male pattern baldness, amenorrhea, clitoromegaly) [3]. Occasionaly patients have estrogenic manifestations (menometrorrhagia, postmenopausal bleeding) [4]. Abdominal mass, pain and distension and ascites are the clinical presentations in approximately 50 % of these tumors [5].

According to the WHO classification [6] SLCTs are subdivided into well, moderately and poorly differentiated tumors depending on the degree of tubular differentiation of the Sertoli cell component (with poorly differentiated tumors having the least tubular differentiation). Heterologous mesenchymal or epithelial elements are found in up to 20 % of SLCTs and they occur only in moderately and poorly differentiated tumors or tumors with retiform pattern [7]. The prognosis depends on the patient’s age, stage of the tumor and the degree of differentiation with the presence of heterologous elements or retiform pattern being a bad prognostic feature [1].

We report an extremely rare case of moderately differentiated retiform SLCT with extensive owergrowth of heterologous rhabdomyosarcomatous (RMS) component occurring in a postmenopausal patient.

## Case presentation

A 70 – year-old, para 2, was referred to our centre with severe right-sided lower abdominal pain that started a few days prior to her visit. She had gained 4 kg, noticed abdominal distension and occasional dull right-sided lower abdominal pain in the 2 months prior to admission. She was otherwise healthy, without any regular medical therapy. She had two vaginal deliveries and her last period was at the age of 46. She had no significant gynecological history. Speculum examination revealed normal-looking vagina and cervix. Upon bimanual palpation a large, smooth and mobile tumor formation, extending from the top of a normal sized uterus up to the umbilicus, was felt. Transvaginal ultrasound (TVUS) examination showed a large, well-vascularized, pelvic mass with solid and cystic components above the uterus and a small amount of ascites in the pouch of Douglas. Contrast enhanced computed tomography (CT) scan of the abdomen showed a well circumscribed, multilocular, complex tumor formation measuring 19 × 12 × 21 cm that appeared to originate from adnexal area, and a thickened, 15 mm, non-homogenous endometrium. Chest X ray showed no evidence of pulmonary nodules. Laboratory tests showed increased serum level of CA-125 at 135.5 U/ml whereas CA 15–3, CA 19–9, CEA were within the normal range. Serum testosterone levels were not determined as there were no signs of androgen excess and they are not a part of a routine pre-operative evaluation at our clinic. Subsequently, exploratory laparotomy was performed. Right ovary with the tumor was removed and sent for intra-operative frozen section evaluation, which revealed features of ovarian malignancy. Hysterectomy with left salpingo-oopherectomy, appendectomy, omentectomy, peritoneal biopsies, pelvic and para-aortic lymphadenectomy were therefore performed and there was no macroscopic disease remnant at the end of the procedure.

On gross examination the tumor was cystic and solid with extensive areas of necrosis and hemorrhage.

**Fig. 1:**
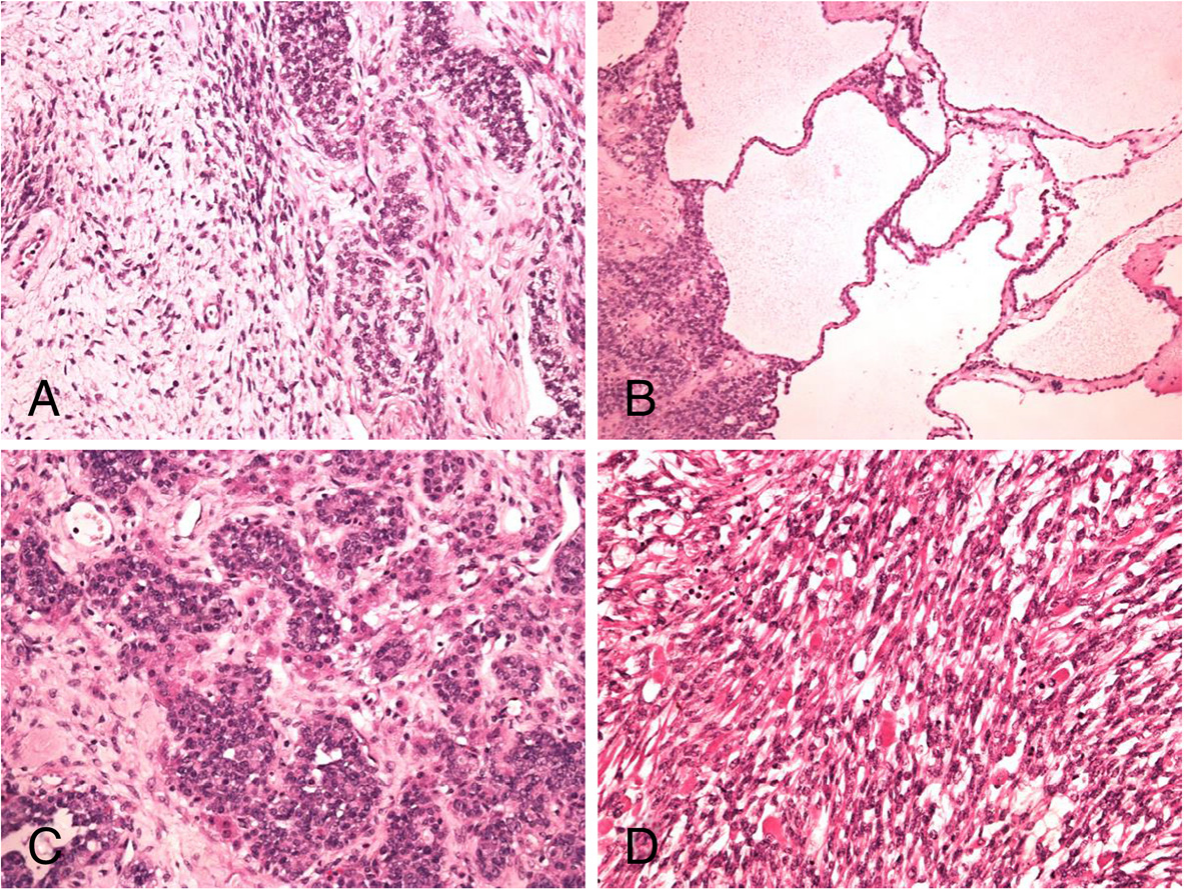
**a** Microscopically, the tumor was composed of a Sertoli-Leydig cell and rhabdomyosarcomatous component (x100). **b** In some areas, dilated cystic structures were present (x100). **c** Sertoli-Leydig cell component composed of cords and islands of Sertoli cells surrounded by Leydig cells with more abundant eosinophilic cytoplasm (x200). **d** Sarcomatous component with many larger cells with abundant eosinophilic cytoplasm suggesting rhabdomyoblastic differentiation (x200)

**Fig. 2:**
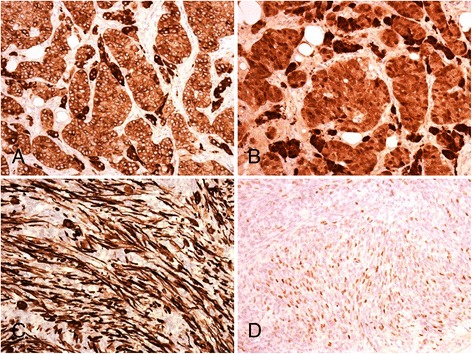
Immunohistochemistrty revealed diffuse strong positivity for inhibin (**a**) and calretinin (**b**) in the Sertoli-Leydig cell component and for desmin (**c**) and myogenin (**d**) in the sarcomatous component (x200)

Microscopically, most of the tumor consisted of a high grade spindle cell sarcoma with numerous mitoses, areas of necrosis and focal cells with ample eosinophilic cytoplasm, consistent with rhabdomyoblasts. Immunohistochemically, this component was diffusely positive for desmin and myogenin. At the periphery of this mass the tumor was composed of cords and nests of cells with clear cytoplasm intimately admixed with cells with more abundant eosinophilic cytoplasm; some cystically dilated structures were also present. Immunohistochemically, both types of cells were diffusely positive for inhibin and calretinin. A diagnosis of a Sertoli-Leydig cell tumor with retiform areas and heterologous rhabdomyosarcomatous component overgrowth was rendered. Morphological and immunohistochemical characteristics of the tumor are depicted in Figs. 1a-d and 2a-d.

The tumor was staged IA and we decided not to administer postoperative chemo- or radiotherapy.

**Fig. 3:**
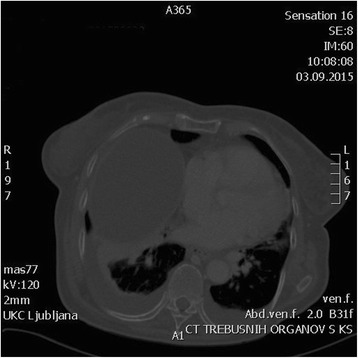
CT scan of recurrent tumor

At her first post-operative check up, 8 weeks post procedure, clinical exam and TVUS showed no signs of disease recurrence and the patient was well. She returned to our clinic 7 months after the procedure with abdominal pain, ascites and a large palpable abdominal mass. She lost 5 kg in 1 month and noticed leg swelling 2 weeks before the admission. CT scan showed a large tumor mass extending from the pelvis measuring 21 × 16 × 20 cm (Fig. 3). Additional tumor masses measuring 10 × 15 × 15 cm below the left hemi-diafragm reaching to the spleen and a mass measuring 3 × 4 × 5.6 cm at the splenic hilum were seen. Serum testosterone level was slightly elevated at 1.7 nmol/L. Furthermore, CT scan showed approximately 2 l of ascites and a massive pulmonary embolism with pulmonary infarction and right-sided heart failure. The patient was treated with therapeutic dosage of low-molecular weight heparin and received several blood transfusions. Nephrostomy tube was inserted because of the right-sided hydronephrosis. Due to the widespread of the disease with poor prognosis and patient’s poor general condition, we decided not to perform the surgical procedure. She received paliative care. She died on the fifteenth day after the admission. Her family resigned from an autopsy.

## Discussion

SLCTs are mixed sex cord-stromal tumors composed of variable proportions of Sertoli cells, Leydig cells and in the case of moderately and poorly differentiated neoplasms primitive gonadal stroma and sometimes heterologous elements. When the tumor shows pattern wich can vary from anastomosing, slit-like spaces resembling rete testis to areas of papillary or multicystic pattern with sieve-like spaces, the term retiform SLCT is used. Retiform pattern is found in approximately 15 % of SLCTs [8]. Retiform SLCTs occur at a younger age (mean age 15 years) and mostly present with a large abdominal tumor and pain rather than virilization [9]. Approximately 20 % of SLCT contain heterologous elements [1, 2, 7]. Heterologous elements include epihelial and/or mesenchymal tissue and tumors arising from these tissues (mucinous gastro-intestinal epithelium, hepatocytes, skeletal muscle, cartilage, adipose tissue, mucinous carcinoma, carcinoid, rhabdomiosarcoma, neuroblastoma) [6]. The most common heterologous element is mucinous epithelium of enteric type, others are rare [1, 2]. Five percent of SLCTs contain mesenchymal elements [10]. Both retiform and heterologous elements are only found in moderately and poorly differentiated SLCTs.

Little is known about the pathogenesis of ovarian SLCT however, germ-line mutations in the microRNA processing gene *DICER1* have been shown to be related with the development of benign tumors that are susceptible to malignant transformation [11]. These tumors include ovarian SLCT, multinodular goiter, multilocular cystic nephroma and pleuropulmonary blastoma [12–14]. Heravi-Moussavi et al. [12] report a 60 % prevalence of *DICER1* mutations in SLCT. Similarly, in the study of Conlon et al. [15] *DICER1* mutations were present in 63 % of SLCT. They also report that there is no association between *DICER1* mutation and tumor differentiation as mutations in poorly differentiated and moderately differentiated SLCT were found to have similar frequencies. It has been established, that reduced expression of *DICER1* is associated with a poor cancer outcome [16]. The exact mechanism underlying tumorigenesis associated with *DICER1* mutations remains to be determined, however.

The prognosis of SLCTs is overall favorable and depends on the stage and histological grade of the tumor and the age of the patient. The overall 5- year survival rate for stage I is 95 % and for stages III and IV almost 0 %. Histological grade also correlates with prognosis as a study of 207 cases of SLCTs reported that well-differentiated tumors behaved benign whereas 11 % of moderately differentiated, 19 % of those with heterologous elements and 59 % of poorly differentiated were clinically malignant [1]. The presence of a retiform pattern seems to have an adverse affect on the prognosis, however, the malignant potential of this pattern remains uncertain [9, 17]. Also, the presence of heterologous mesenchymal elements appears to be associated with a poor prognosis [18].

Because SLCTs are very rare, a standardized approach for treatment has not been established yet. Unilateral salpingo-oopherectomy seems to be sufficient in well-differentiated unilateral SLCTs [19]. In cases of moderately and poorly differentiated SLCTs and SLCTs with heterologous elements hysterectomy, bilateral salpingo-oopherectomy and staging (omentectomy, appendectomy and pelvic lymphadenectomy) should be performed [20]. Maximal tumor debulking is recommended for SLCTs with extra-ovarian spread. Adjuvant chemotherapy has been suggested for cases of stage IB to IV, recurrent SLCTs and poorly differentiated SLCTs with heterologous elements [21] however, the value of adjuvant chemotherapy has not yet been determined [22]. Grove et al. [23] suggested that for moderately differentiated SLCTs with heterologous elements, the percentage of sarcoma and its cellular differentiation should be evaluated to decide whether or not to use adjuvant chemotherapy. Their patient had a moderately differentiated SLCT with RMS elements and did not receive postoperative chemotherapy. Despite that, she was disease free 4 years after the procedure. On the other hand, Prat et al. [18] suggested that in cases of SLCTs with mesenchymal elements the prognosis is as poor as in primary ovarian sarcomas and thus adjuvant chemotherapy should be given for all disease stages.

In our case, a moderately differentiated retiform SLCT with retiform areas and overgrowth of the heterologous RMS component occurred in a postmenopausal patient. Two of the previously described cases of moderately differentiated SLCTs with RMS elements occupying only small parts of the tumor had a benign disease course [23, 24]. In contrast, a case where the RMS component almost overgrew the SLCT, had a malignant disease course with recurrence 6 and 10 months post surgical therapy [25]. The recurrent tumor was composed exclusively of RMS elements. Brief summarization of cases of SLCTs containing malignant heterologous mesenchymal elements can be found in Table 1.

**Table 1:** Presentation of case reports of SLCTs containing malignant heterologous mesenchymal elements

Author	Patient’s age	Tumor size	Laterality	FIGO stage	Treatment	SLCT grade	Heterologous elements	Recurrence site	Recurrence treatment	Outcome
Prat et al. 1982 [18]		13–22 cm								
	23		Right ovary	IA	Right SO	/	Immature skeletal muscle + Immature cartilage + Gastrointestinal epithelium	Peritoneum, Pelvis	Chemotherapy	Alive with tumor after 2 years
	24		Not specified	IA	Unilateral SO	/	Immature cartilage	Omentum, Pelvis	Surgery, Chemotherapy	Died after 18 months
	14		Left ovary	IA	Left SO	Poor differentitation	Cartilage in recurrent tumor	Pelvis	Radiotherapy	Died after 9 months
	16		Right ovary	IA	Right SO	/	Immature skeletal muscle	Left ovary, Peritoneum	TAH + Left SO; Chemotherapy	Died after 7 years
	20		Not specified	IIA	TAH + Bilateral SO	Poor differentiation	Skeletal muscle in recurrent tumor	Peritoneum	Surgery, Radiotherapy, Chemotherapy	Died after 10 months
	36		Right ovary	IIB	Right SO	/	Immature skeletal muscle + Immature cartilage	Peritoneum	/	Died after 6 months
	48		Left ovary	IA	TAH + Left SO	/	Immature skeletal muscle + Immature cartilage	Not specified	Not specified	Not specified
	20		Right ovary	IIB	Right SO + Chemotherapy	/	Immature skeletal muscle	Left ovary, Douglas pouch	TAH + Left SO; Radiotherapy	Died after 18 months
	32		Right ovary	IA	TAH + Right SO	/	Immature skeletal muscle	Peritoneum	/	Died after 5 months
	22		Not specified	Not specified	Biopsy	/	Immature skeletal muscle	Not specified	Not specified	Not specified
	17		Left ovary	IC	Left SO	/	Immature skeletal muscle	Right ovary, Pelvis	TAH + Right SO Omentectomy	Died after 12 months
	11		Left ovary	IA	Left SO	/	Immature cartilage	/	/	Alive after 10 years
Kostopolou, Talerman, 2003 [24]	22	6 cm	Left ovary	IA	Left SO	Moderate differentitation	Immature skeletal muscle	/	/	Disease free after 10 months
Grove et al. 2006 [23]	29	18 × 14 × 11 cm	Right ovary	IC	Right SO + Omentectomy	Moderate differentitation	Embrional rhabdomyosarcoma + Immature cartilage	/	/	Disease free after 48 months
Guerard, Ferenczy, 1982 [25]	16	20 × 20 × 10 cm	Left ovary	IC	Left SO	Moderate differentitation	Pleomorphic rhabdomyosarcoma	Abdomen	Surgery, Chemotherapy	Recurrence after 6 and 10 months

*SO* salpingo-oopherectomy, *TAH* total abdominal histerectomy

Our patient had a FIGO stage IA tumor at the time of the surgical treatment and complete macroscopic cytoreduction was achieved. Despite that, her disease recurred in 7 months confirming the aggressive behavior of SLCT containing RMS and/or retiform elements. We assume that the recurrent tumor was mainly composed of RMS elements due to the aggressive disease course and only slightly elevated serum testosterone levels. The rapid disease progression implies that the presence of RMS and/or retiform component in primary SLCTs causes an unfavorable disease course and that it is the heterologous component in the SLCTs that affects the prognosis and not the grade of the Sertoli – Leydig component itself, as it has already been suggested [1]. However, the incidence of moderately differentiated SLCTs with RMS and SLCTs with retiform component is very low, and we are not able to conclude how these elements truly affect the disease course.

Ovarian RMS is an extremely rare tumor with a very aggressive disease course and poor outcome regardless of treatment [26]. It has been shown that optimal cytoreduction to no gross residual disease improves survival outcome in ovarian sarcomas [27, 28] and that age of the patient and stage of the tumor are associated with overall survival as well [28]. On the other hand, there are no established guidelines whether patients with these tumors should receive postoperative chemotherapy and which regimen would be optimal. Bacalbasa et al. [29] report of a case of primary ovarian RMS where the patient had a disease recurrence 2 months post surgical treatment and died 4.5 months after the second surgical intervention despite receiving adjuvant chemotherapy.

There are no specific guidelines about the follow-up of patients after the surgery for SLCTs. In our case, the patient was followed up as patients with epithelial ovarian cancer. Despite the fact that at her first post-operative check-up, when clinical and TVUS examination were performed, she was disease free, the recurrent tumor 6 months later was widespread and therefore considered as not suitable for surgical treatment. Thus, we believe that imaging examination with a higher specificity and sensitivity should be used for detection of SLCTs recurrence. Recently, positron emission positron emission tomography (PET) combined with computed tomography (CT) has been proposed as an imaging modality for early detection of recurrence of ovarian cancer [30]. Gu et al. [31] reports of a 93 % pooled specificity and 91 % pooled sensitivity of PET CT for detection of recurrence of ovarian cancer. It has not yet been established however, whether it would have similar success detecting SLCT recurrence. Furthermore, there are no guidelines on when to start and how often to use PET-CT in patient follow-up after the treatment.

## Conclusion

Management of ovarian SLCTs with malignant disease course is challenging due to the rarity of the disease and lack of experience. It seems that overgrowth of the SLCTs with RMS elements causes a very unfavorable disease course. Further reports of individual cases as well as randomized controlled studies are needed for establishment of management guidelines and the role of adjuvant chemotherapy in these tumors. At present, there is no good evidence that postoperative adjuvant chemotherapy is effective in preventing recurrence of malignant SLCTs. Toxicity and the ability to complete the chemotherapy regimen should be kept in mind when deciding about application of toxic medications.

## Abbreviations

CT, comuted tomography; PET, positron emission tomography; RMS, rhabdomyosarcoma; SLCT, Sertoli Leydig cell tumor; TVUS, transvaginal ultrasound
